# Synthesis and Photophysics Characterization of Boronic Styril and Distyryl BODIPYs for Water-Based Dye-Sensitized Solar Cells

**DOI:** 10.3390/biomimetics7030110

**Published:** 2022-08-11

**Authors:** Francesco Nastasi, Placido Giuseppe Mineo, Jessica Barichello, Giuseppina La Ganga, Gaetano Di Marco, Giuseppe Calogero, Massimiliano Cordaro

**Affiliations:** 1 Department of Chemical, Biological, Pharmaceutical and Environmental Sciences, V. le F. Stagno D’Alcontres 31, University of Messina, 98166 Messina, Italy; 2 Department of Chemical Sciences, University of Catania, V. A. Doria 6, 95100 Catania, Italy; 3 CNR-IPCF, Institute for Chemical-Physical Processes, Via F. Stagno D’Alcontres 37, 98158 Messina, Italy; 4 CHOSE—Center for Hybrid and Organic Solar Energy, Department of Electronic Engineering, University of Rome “Tor Vergata”, 00133 Roma, Italy; 5 CNR-ITAE, Istituto di Tecnologie Avanzate per l’Energia, 98126 Messina, Italy

**Keywords:** BODIPY, dye-sensitized solar cells, boronic acids, external quantum yield, donor-π-acceptor

## Abstract

In this study, two boronic acid BODIPYs are obtained through a microwave-assisted Knoevenagel reaction. The aim is to use them for the first time as dyes in a photosensitized solar cell (DSSC) to mimic chlorophyll photosynthesis, harvesting solar light and converting it into electricity. The microwave-assisted Knoevenagel reaction is a straightforward approach to extending the molecular conjugation of the dye and is applied for the first time to synthesize BODIPY’s boronic acid derivatives. These derivatives have proved to be very useful for covalent deposition on titania. This work studies the photo-physical and electrochemical properties. Moreover, the photovoltaic performances of these two new dyes as sensitizers for DSSC are discussed. Experimental data show that both dyes exhibit photosensitizing activities in acetonitrile and water. In particular, in all the experiments, distyryl BODIPY was more efficient than styryl BODIPY. In this study, demonstrating the use of a natural component as a water-based electrolyte for boronic BODIPY sensitizers, we open new possibilities for the development of water-based solar cells.

## 1. Introduction

Dye-sensitized solar cells (DSSCs) are photovoltaic (PV) devices that employ different kinds of coloring substances for sunlight harvesting [1]. Since their official appearance in 1991 [2], DSSCs have drawn a lot of attention in the scientific community due to their ease of fabrication, the chance to apply natural components that will lower the production cost [3,4,5] and their high PV performance under weak light intensity in comparison to other *p–n*-junction-based photovoltaics [4,6,7]. DSSC technology is extremely interesting from an esthetic perspective; moreover, the capacity to filter light using the color tunability of the selected dye makes DSSC competitive with other PV technologies in an indoor environment [8,9]. Recently, a new era of DSSC industrialization has been proposed for niche applications and low-power devices such as remote controls for projectors and Internet of Things (IoT) sensor systems [1].

A typical DSSC is assembled by placing a transparent photo-anode (or working electrode, WE) and a counter-electrode (CE), with an electrolyte solution in the middle, to promote charge transfer via a redox mediator (usually an iodide/iodine redox couple, I^−^/I_3_^−^, although other redox mediators have been successfully tested). Numerous technologies aim to mimic the perfect natural photosynthesis system, which involves light-energy absorption and charge separation [10]. In a DSSC, the dye substitutes the chlorophyll molecule in harvesting photons, while the liquid electrolyte regenerates the dye as water in the same way that a biological system regenerates chlorophyll. Generally, the photoanode consists of a dye-sensitized mesoporous wide-band semiconductor layer (TiO_2_, ZnO, SnO_2_) deposited on a transparent conductive glass substrate. In the light, dye molecules capture the incident photons, generating electron/hole pairs. The resulting electrons, at excited states, are then injected into the conduction band of the semiconductor oxide and transported to a CE that transfers them to the electrolyte. The electrolyte reduces the oxidized dye, which is reported to the ground state, ready to absorb another incident photon, and the cyclic process is repeated several times. Traditional liquid electrolytes are prepared, including an organic solvent (acetonitrile, butirronitrile, etc.). These have the relevant drawbacks of high vapor pressure and severe environmental impacts. Moreover, several organic solvents are dangerous, meaning that their practical applications in DSSCs are seriously limited due to safety issues. Regarding the solvents that can be employed inside the DSSC, some researchers decided to return their attention to the water, the solvent of life. Indeed, employing water-based electrolytes reduced costs, removed flammability, reduced volatility and improved environmental compatibility [11]. Despite the numerous benefits that can be obtained using water-based electrolytes, the detachment of dye molecules from the semiconductor surface is a common limitation. Indeed, water and dye molecules compete for adsorption on the hydrophilic surface of the semiconductor [12]. Therefore, the use of hydrophobic dyes with a water-based electrolyte is mandatory. Different research groups have recently demonstrated that a low amount of water (5–20%) in the electrolyte composition decreases the TiO_2_ lattice disorder, improving the photo-current density [13,14,15]. The incorporation of a small amount of water works as a doping strategy, incorporating oxygen from water into TiO_2_ oxygen vacancies, leaving hydrogen atoms exposed on their surface; this results in a band-edge shift toward more positive potentials due to the positive charge accumulation at the TiO_2_ surface. This results in a more efficient electron injection in the TiO_2_ conduction band, causing a gradual increase in the current. For the same reason, a progressive open-circuit voltage reduction is observed in the downward shift (toward positive potential) of the TiO_2_ band edge. A PCE of 4.47%, one of the highest reported, was obtained with a gelled aqueous electrolyte in a quasi-soli state DSSC. Moreover, it showed stability during 5 days of ageing [16]. The first completely natural DSSC was realized with chlorophyll pigments as dye and a water-based electrolyte, but obtained poor results in terms of PCE [17].

By using water as a 100% electrolyte solvent, we became closer to achieving the initial purpose of DSSC inventors, using photosynthesis as the inspiration for the working system. Moreover, the sustainability of the developed DSSC device will improve by using only water as solvent for the electrolyte solution. Despite this, and the change to the use of natural pigments as dyes that increase the greenness of the PV technology, some sustainability issues are still present: the TCO/glass substrate is a highly energy-demanding material and its waste management leads doubts regarding its feasibility. Further concerns include the employment of noble metals as cobalt as redox couple in the electrolyte, and the use of platinum and ruthenium [18]. The dye is an essential component of a DSSC, and many natural and artificial sensitizers have been investigated [1,4,5]. While considerable research efforts have been directed to the study of DSSCs, the remaining challenge is to improve dye chemical stability and its anchorage to the semiconductor surface.

**Figure 1:**
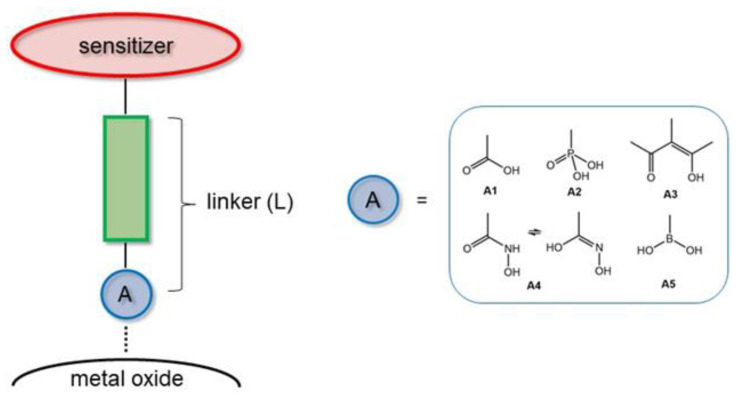
Linker molecules (L) are replaced with different anchoring groups A: carboxylic acid (A1), phosphonic acid (A2), acetyl acetonate (A3), hydroxamic acid (A4), boronic acid (A5).

It is essential to study the electronic and redox properties of the sensitizer and it is no less important to find the best photo-induced transfer efficiency by studying the linker structure/functionality (Figure 1). These studies show that linkers with highly conjugated molecular structures transfer electrons more rapidly than aliphatic linkers [19,20,21].

All the anchor groups contain functionalities that can form ester bonds with metal oxides. These bonds are not always very stable, as they are generally pH-sensitive and thermally labile [22]. The use of carboxylic-acid-based systems is the most popular strategy in the literature, owing to their relative ease of preparation and exceptional performance with respect to electron injection. On the other hand, the boronic acid demonstrates a strong and stable bind on the anatase surface, since boron has different electron negativities regarding carbon, which increases the stability and strength of the covalent bond between boronic acid and anatase surface [23]. The use of boronic acids offers a valid alternative to the use of carboxylic acid and phosphonic acid functionalities, showing several advantages:

(a) The boronic derivatives are commercially available for their extensive use in organic synthesis as precursors in the C-C coupling reactions [24];
(b) The insertion procedures of boronic groups are widely reported in the literature [25,26];
(c) Boronic derivatives show generally high stability and moderate toxicity [27];
(d) Boronic acid derivatives form very stable esters [28].

**Figure 2:**
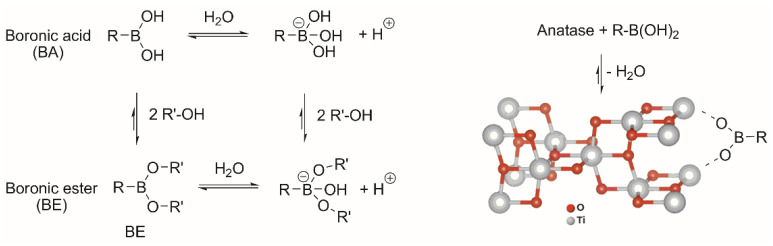
pH-dependent equilibrium of boronic derivatives (BA and BE) and plausible interaction between boronic acid and TiO_2_ surface (anatase).

Boronic acids (BA) are able to react with various nucleophiles since the boron center can reversibly coordinate with the hydroxyl groups to form stable boronate esters (BE). In the aqueous phase, many studies have been performed to define the mechanism and stability of boronic esters, but the factors that govern these processes are still the subject of considerable debate. It has been hypothesized that the tetrahedral boronate anion favors complexation at higher pH values (Figure 2). The BE stability is highly dependent on the structure of the alcoholic ligand; typically, cis-1,2 cyclic diols generate more stable complexes. Over the years, BE has played a significant role in building hybrid materials for a multitude of applications [29].

Usually, TiO_2_ is employed due to its wide conduction band and *d* orbital [30]. Many organic chromophores have been studied to find a substitute for organometallic complexes [31,32]. To date, the overall conversion efficiency of these sensitizers has always been lower than the ruthenium-complex dyes, although the lower costs of their preparation make them highly competitive. Among the latest generation of organic dyes, boron dipyrromethene (BODIPY) systems are the most versatile in all fields of application [28,33,34], and have an advantage due to the unlimited number of procedures that can be used for their derivation and the ad hoc modification of their chemical–physical properties. Thus, the BODIPYs find applications as chemosensors [35,36], logic gates [37], light collectors [38,39], energy transfer cassettes [40,41] and photodynamic therapeutic agents [42,43].

We believe that BODIPY dyes are promising, if properly designed, and highly advantageous in various applications compared to most other organic dyes [44]. One of the first main studies on the use BODIPY as a sensitizer for TiO_2_ nanocrystalline mesoporous electrode was pioneered by Hattori et al. [45]. BODIPY dyes have inherent asymmetry in charge redistribution when they undergo S_0_→S_1_ transition upon excitation, increasing the charge density on the meso-carbon (C-8), while decreasing it in most other positions in the boradiazaindacene. The directionality observed in excitation can be further enhanced with strategically placed electron-withdrawing and electron-donating groups [46].

This study aims to analyze the photophysical and photochemical properties of two new BODIPY dye derivatives (the non-symmetrical monostyryl **1** and the symmetrical distyryl **2**), which are used as sensitizers for DSSCs (Figure S1). These new BODIPY dye derivatives are featured in boronic acid groups as anchoring units and a nitro-phenyl moiety, which is useful for future chemical reactions. The obtained molecules have been characterized by NMR (Figures S2–S9) and mass spectrometry analyses (Figure S10). Their photophysical behaviours were studied using UV-Vis absorption and fluorescence emission spectroscopies. The new dyes were synthesized according to a classic synthetic strategy and Knoevenagel condensation reaction promoted by microwave (MW) irradiation [47]. Finally, we tested the two new dyes in DSSC devices with two electrolytes: the most common acetonitrile is based on the I^−^/I^3−^ redox couple and in a water solution with a quinone/hydroquinone (Q/H_2_Q) redox couple. The Q/H_2_Q redox couple is important in biological redox processes [48]; therefore, we selected this for use in a complete water-based electrolyte. To our knowledge, this is the first study that shows a water-based BODIPY DSSC system and successfully demonstrates that this system is also able to convert light energy in electrical energy.

## 2. Results and Discussion

**Figure 3:**
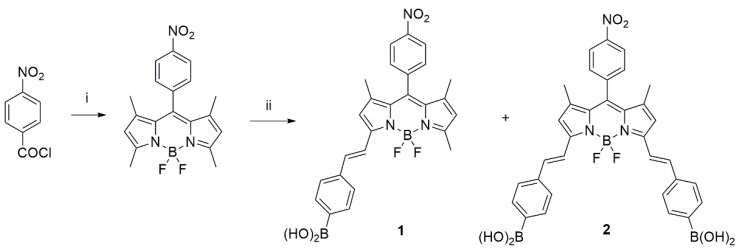
Synthetic route for monostyryl boronic acid-BODIPY **1** and distyryl boronic acid BODIPY **2**: (**i**) 1. 2,4-dimethylpyrrole, dichloromethane anhydrous, argon, r.t., 2. triethylamine, 3. boron trifluoride diethyl etherate. (**ii**) 4-formylphenylboronic acid, piperidine, acetic acid, toluene anhydrous, MW 900W, reflux, 6 h.

BODIPY dyes **1** and **2** were synthesized using a one-pot procedure, starting from 4-nitrobenzoyl chloride and pyrrole and a subsequent BF_2_ insertion after basification with trimethylamine (scheme in Figure 3). The formation of the styryl bonds does not occur by common thermal heating; microwave-assisted synthesis was used. The Knoevenagel condensation is difficult, probably due to the formation of boroxine [49].

**Figure 4:**
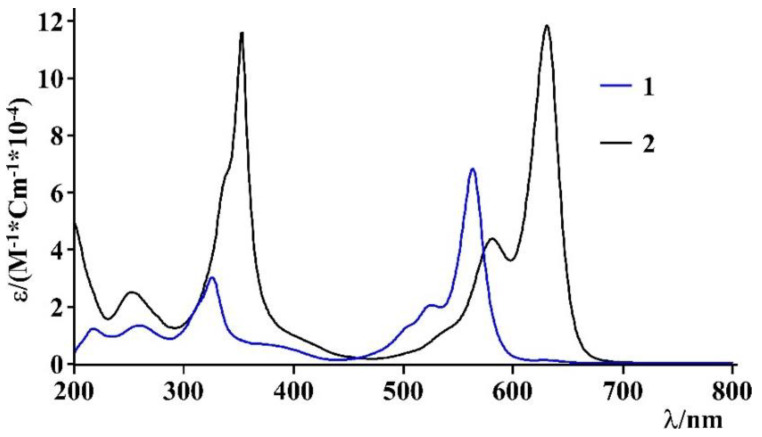
UV-vis absorption spectra of BODIPY **1** (blue line) and BODIPY **2** (black line) in acetonitrile.

The absorption spectra of BODIPY **1** and BODIPY **2** in acetonitrile are shown in Figure 4.

In particular, these systems show intense absorption bands, **which are** typical of BODIPY chromophore subunits, assigned at spin-allowed π-π* transitions, with a maximum at 564 nm (ε = 67,900 M^−1^ cm^−1^) for the BODIPY **1** and at 633 nm (ε equal to 116,000 M^−1^ cm^−1^) for the BODIPY **2**. The main photophysical properties are reported in Table 1.

All the investigated species show interesting luminescence properties with intense emission bands, in both solution (at room temperature) and a rigid matrix (at 77 K).

**Figure 5:**
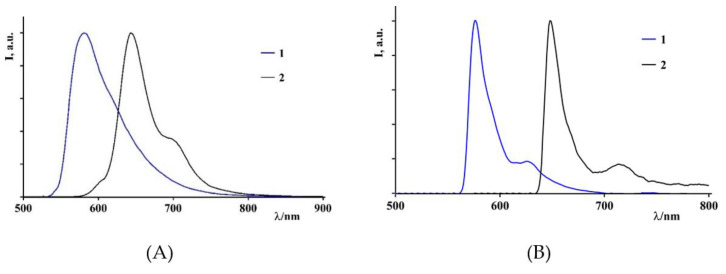
(**A**) Emission spectra of BODIPY **1** (blue line) and BODIPY **2** (black line) in acetonitrile at 298 K. (**B**) Emission spectra of BODIPY **1** (blue line) and BODIPY **2** (black line) in butyronitrile at 77 K.

The luminescence is attributed to fluorescence from the BODIPY singlet state [50]. The emission spectrum of the BODIPY **1** at room temperature is dominated by a band with a maximum of 580 nm, regardless of the wavelength used for the excitation, with a quantum emission yield of 0.008 and a state lifetime excited by 0.8 ns. At 77 K in the rigid matrix, the emission spectrum is dominated by an intense band centered at 575 nm with a lifetime of the excited state of 4.3 ns. For the BODIPY **2**, the emission spectrum, at room temperature, is dominated by a band with a maximum of 650 nm, regardless of the wavelength used for the excitation, with a quantum emission yield of 0.1, and the lifetime of the excited state of 1.8 ns. At 77 K in the rigid matrix, the emission spectrum is dominated by an intense band centered at 645 nm with an excited-state lifetime of 4.0 ns, (Figure 5A,B and Table 1).

This spectroscopic behavior is typical of BODIPY with a nitrobenzene substituent, where an electron-deficient benzene subunit could quench the luminescence of the BODIPY via a photo-induced electron transfer process [51]. The electrochemical measurements were carried out using BODIPY **1** and **2** (C = 1 × 10^−4^ M) solutions in acetonitrile, with TBA as electrolyte support. The obtained dta are summarized in Table 2. Differential pulse voltammograms of BODIPY 1 and 2 are reported in the Supporting Information (Figures S11 and S12).

**Figure 6:**
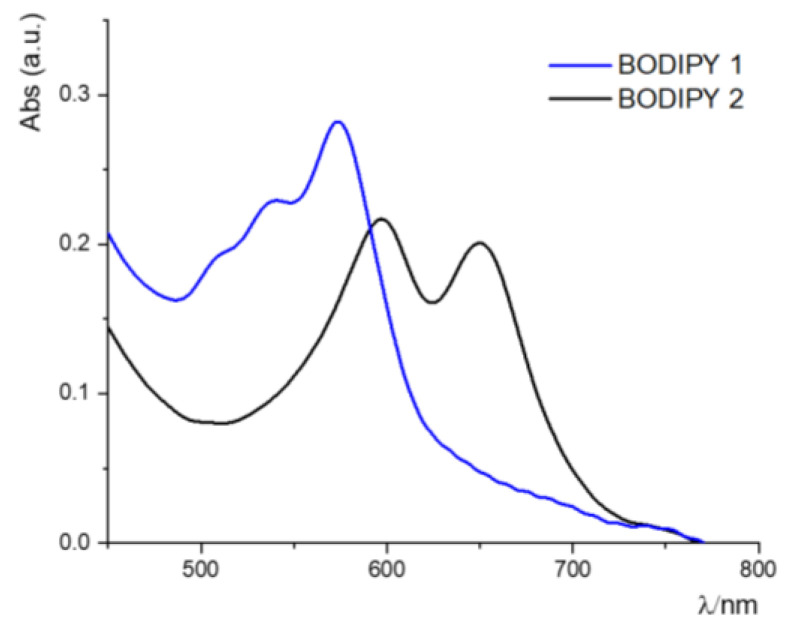
Absorption spectra of BODIPY **1** (Blue line) and BODIPY **2** (Black line) anchored on TiO_2_.

The BODIPY **1** exhibits a monoelectronic and quasi-reversible oxidation process at +1.04 V vs. SCE and a monoelectronic and quasi-reversible reduction process at −0.903 V vs. SCE. The BODIPY **2** exhibits a monoelectronic and quasi-reversible oxidation process at +0.959 V vs. SCE and a monoelectronic and quasi-reversible reduction process at −0.857 V vs. SCE. As far as redox properties are concerned, the values of the potentials of both BODIPYs under investigation, when compared with the data reported in the literature [52], allow the reduction of the nitrobenzene fragment to be assigned to the first process observed in the cathodic region, while the first oxidation process can be assigned to the BODIPY fragment. To verify the performance of these BODIPYs as sensitizers for DSSC, photo-anodes were made and DSSC devices were assembled. Based on the photo-physical data and the redox properties, it is possible to hypothesize that a photo-induced electron-transfer process can take place in the devices, involving the excited state of the BODIPY as a donor and the TiO_2_ layer as an electron acceptor. From the combination of the experimental data summarized in Table 1 and Table 2, it is possible to estimate the driving force of the electronic transfer process, considering the following relationship:

(1) $$\Delta G{}^{\circ}=\left(E_{OX}^{*}-E_{RED}\right)$$

(2) $$E_{OX}^{*}=E_{OX}-E_{00}$$

where E_OX_ is the oxidation potential of BODIPY, E_RED_ is the reduction potential of TiO_2_ nanoparticles, whose value can be estimated with good approximation at −0.40 V vs. SCE [48], E_00_ is the potential corresponding to the excited luminescent state. Emission spectra carried out at 77 K confirm that the energy levels of LUMO have very similar energy values to those of the TiO_2_ conduction band, which justifies the small electron injection efficiencies for the two sensitizers. The driving force for the electronic transfer process is equal to −0.716 eV for the BODIPY **1** and −0.563 eV for the BODIPY **2**, indicating that the TiO_2_ photo-injection process is thermodynamically allowed. Once the photo-electrodes have been made, the first step was to check their stability by washing them using aqueous solutions at a different pH (3 < pH < 7) and organic solvents such as acetonitrile, acetone and ethanol. The obtained photo-anodes were characterized by UV-Vis spectroscopy. The absorption spectra of the electrodes are shown in Figure 6.

It is possible to observe that, even if assembled on the surface of the electrode, the chromophores have intense absorption bands, which are, however, less structured and wider than the species in solution (Figure 4). This behavior is attributable, by comparison with the literature data [53], to the aggregation phenomena on the surface. From the absorption spectra, we estimated the dye loading by the Lambert–Beer law [23,54,55]. For BODIPY 1 and BODIPY 2, we found a dye-loading concentration of 3.82 µM and 1.47 µM respectively. The higher absorption of BODIPY 1 in comparison to BODIPY 2 is attributable to the one boronic chain with which BODIPY 1 anchors on the TiO_2_ surface, occupying a smaller surface area than BODIPY 2 that anchors with both the boronic chains. After checking the high adsorption capacity and stability on TiO_2_ film, their properties as photosensitizers in DSSC systems were verified using the iodide I^−^ and triiodide I_3_^−^ pair as a RedOx mediator, without the presence of any basic compounds and using acetonitrile as a solvent.

The performance of a solar cell is characterized by five main parameters: incident photon-to-current conversion efficiency (IPCE), open-circuit voltage (V_OC_), short-circuit photocurrent density (J_SC_), fill factor (FF) and solar energy-to-electricity conversion yield (PCE). For each dye, BODIPY 1 and 2, three cells were realized and, in Table 3, the PV parameters of the best-obtained device are reported. The PV parameters of other devices are reported in Table S1.

Under irradiation (AM 1.5G, 95 mW × cm^−2^) the device obtained using BODIPY **1** as the dye generates a photocurrent density of 0.73 mA/cm^2^ with a V_OC_ of 0.267 V and an efficiency of 0.11% (see Figure S13 and Table 3). Under the same conditions, the device using BODIPY **2** as a sensitizer shows better activity as a photosensitizer, generating a photocurrent density of 3.1 mA/cm^2^ under irradiation, with a V_OC_ of 0.377 V and an efficiency of 0.63% (see Figure S14 and Table 3). For both the devices, the incident photon-to-current conversion efficiency (IPCE) with the integrated current density (I-Jsc) were recorded in a range of wavelengths between 400 and 750 nm (see Figure S15 and Table 3). The BODIPY **1** device shows constant values of IPCE of around 4%, while the BODIPY 2 device **2** shows a maximum IPCE of 24% at 650 nm. The integrated current density (0.78 and 2.7 mA/cm^2^ for BODIPY 1 and 2, respectively) has a slight and negligible mismatch and I-Jsc values confirm the goodness of PV performances under the sun simulator. The analysis of these data shows that both compounds have moderate photosensitizer properties. One plausible reason for the moderate PCE values of BODIPY sensitized solar cells could be the existence of two F− ions in the BODIPY core structure. The strong electron-withdrawing F atoms may slow the energy injection rate. Furthermore, although the driving force for the BODIPY **1** is greater than that of the BODIPY **2**, the devices obtained with the latter appear to have a better performance. This effect is attributable to the double anchoring allowed by the boronic functions of the BODIPY **2**, which, by preventing free rotation around the phenyl groups, increases the electronic coupling between the excited sensitizer and the TiO_2_ conduction band [56].

Finally, based on the high stability of the photoanodes obtained with these compounds, their efficiency was also verified in water at pH 7 using the hydroquinone/hydroquinone (Q/H_2_Q) pair as a RedOx mediator [57]. The obtained data are summarized in Table 4. In Table S2, we report the results of an extra fabricated cell for each dye.

Even under these conditions, employing water as a solvent and H_2_Q/Q as a mediator, both compounds exhibit photosensitizing activities. Similarly, to the experiments in acetonitrile, the BODIPY **2** has a higher efficiency, even in water. Furthermore, this is the first time that a BODIPY was employed in water-based DSSCs. The PCE loss using the water substitution as a solvent in a liquid electrolyte is a well-known effect in the literature. One of the main limitations in the use of water as electrolyte solvent is the detachment of the dye molecules from the surface of the semiconductor. Indeed, the water and dye molecules compete for absorbance on the hydrophilic TiO_2_ surface. The absorbed water molecules on the semiconductor surface modify its crystal structure. Researchers have shown that adding less than 5% of water in the electrolyte can increase the PCE, but overcoming the 5% value of water concentration leads to a PCE decrease, which is mainly due to a loss in FF values [13,58].

However, the use of water as a solvent for electrolytes in DSSC is worth investigating in more depth due to its incredible benefits in economics and sustainable terms.

Regarding the photo-physical properties, these systems show intense absorption bands, characterized by a very high molar extinction coefficient and intense emission bands, at both room temperature in a liquid matrix and 77 K in a solid matrix. This luminescence is attributed to the radiative deactivation of the lower-energy excited state. Regarding the redox properties, the values of the potentials of both BODIPYs under examination, if compared with the data reported in the literature, allow for the reduction in the nitrobenzene fragment to be assigned to the first process observed in the cathodic region, while the first oxidation process can be assigned to the BODIPY fragment. The collected experimental data made it possible to evaluate that the photo-injection process of TiO_2_ dyes is thermodynamically allowed. Furthermore, it was observed that both BODIPYs are photosensitizers for DSSC, in both acetonitrile (using the pair I_2_/I_3_^−^ as RedOx mediator) and in water at pH 7 (using the hydroquinone/hydroquinone pair as the RedOx mediator). In particular, devices obtained with BODIPY **2** have a better performance than BODIPY **1**. This effect is attributable to the double anchorage allowed by the boronic functions of BODIPY **2**.

## 3. Materials and Methods

All the employed chemicals were used as received, without any further purification. All NMR spectra were recorded with Varian 500 instrument. Positive ESI mass spectra were acquired on an API 2000 mass spectrometer (ABSciex—Milano, Italy) equipped with an API ion source. The methanolic solutions of the investigated samples were introduced into the source at a flow rate of 5 µL/min. The mass spectra were elaborated with the ‘‘Analyst’’ software (from AB Sciex). The positive MALDI-TOF mass spectra were collected by a Voyager DE (PerSeptive Biosystem). The instrument was equipped with a nitrogen laser (emission at 337 nm for 3 ns) and a flash AD converter (time base 2). To avoid molecule fragmentation, the laser irradiance was maintained slightly above the threshold (approx. 106 W cm^−2^). The MALDI investigations were performed by loading on the plate a 0.1 mmol sample and 40 mmol matrix trans-2-[3-(4-tert-butylphenyl)-2-methyl-2-propenylidene]-malonitrile (DCTB), with DMF as a solvent. The conductive glass plate (FTO glass, fluorine-doped SnO_2_, sheet resistance 7 Ohm/cm^2^) and Surlyn foils 25 μm thick were purchased from Solaronix SA (Aubonne, Switzerland) and used as supplied. The WE absorption spectra were recorded by a Perkin Elmer L20 spectrophotometer UV-Vis (range 180–1100 nm). A digital Keithley 236 multimeter connected to a PC and controlled by a homemade program was used to obtain the current–voltage (I-V) curves for the constructed DSSCs. Simulated sunlight irradiation was provided by an LOT-Oriel solar simulator (Model LS0100-1000, 300 W Xe-Arc lamp, powered by LSN251 power supply equipped with AM 1.5 filter, 100 mW/cm^2^). Incident irradiance was measured with a Si-based pyranometer. Incident photon-to-current conversion efficiency (IPCE or η) and the relative photoaction spectra of sealed DSSCs were measured by an IPCE station consisting of a 150 Xenon Light Source (model ASB-XE, Spectral Products), a Monochromator (model CM110, Spectral Products) equipped with a slit set, a Si-calibrated detector (model 818-UV, Newport), a picoamperometer (model 6487, Keithley) and an IPCE Solarena Software. The DSSCs’ photoanodes thickness was measured using a DektakXT profilometer (Bruker) equipped with a diamond-tipped stylus (radius of 2 µm) and a stylus force of 1 mg. Each measure was verified with the different runs acquired with different start positions by rotating or translating the sample. Emission quantum yields were determined at room temperature using the optical dilute method. As luminescence quantum yield standards, BODIPY **2** of the article in air-equilibrated acetonitrile solution was used (Φ = 0.69) [59]. The absorption spectra of the BODIPYs were recorded using a Jasco V-560 UV/VIS spectrophotometer. Electrochemical measurements were carried out in the dry and argon-purged stated acetonitrile, at r.t., with Autolab multipurpose equipment interfaced to a PC. The working electrode was a glassy carbon (8 mm^2^, Amel) electrode. The counter-electrode was a Pt wire and the pseudo-reference electrode was a silver wire. The reference was set using the redox couple ferrocene/ferrocinium as internal reference (395 mV vs. SCE in acetonitrile). To record the luminescence spectra of the samples, a Spex-Jobin Yvon FluoroMax-2 fluorimeter equipped with a Hamamatsu R3896 photomultiplier was used and corrected for the photomultiplier response with a program supplied with the instrument. An Edinburgh OB 900 “time-correlated single-photon-counting” (TC-SPC) spectrometer was used to determine the lifetimes of the excited states. The sources used for the excitation of the samples are a Hamamatsu PLP 2 diode laser (λ_ecc_ = 408 nm, pulse duration 59 ps) and nitrogen discharge (λ_ecc_ = 337 nm, pulse duration: 2 ns)**.**

### 3.1. Synthesis of 8-(4-Nitrophenyl)-3,5,7,9-Tetramethyl BODIPY

4-nitrophenyl BODIPY was synthesized in a one-pot sequence according to the known procedure [60]. 4-nitrobenzoyl chloride (500 mg, 2.7 mmol) and 2,4-dimethylpyrrole (500 mg, 5.4 mmol) were solubilized in anhydrous dichloromethane (15 mL); the mixture was heated at 50 °C for 1 h in Ar atmosphere. After cooling to room temperature, triethylamine (2 mL, 15.4 mmol) was added to the mixture and stirred for an additional 30 min. Therefore, boron trifluoride diethyl etherate solution (1.5 mL, 12.0 mmol) was added dropwise and the mixture was stirred at room temperature for 12 h. Dichloromethane (50 mL) was added, and three extractions (10% sodium hydrogen carbonate solution, brine and water, respectively) were carried out to remove the inorganic salts. The organic solution was dehydrated with anhydrous sodium sulfate and evaporated. The crude solid was subjected to column chromatography packed with silica as a stationary phase and eluted with dichloromethane. The 4-nitrophenyl BODIPY was isolated as a red–brown solid (350 mg, yield 35%).

^1^H-NMR (500 MHz, CDCl_3_) δ 8.38 (d, J = 8.7 Hz, 2H), 7.54 (d, J = 8.7 Hz, 2H), 6.01 (s, 2H), 2.56 (s, 6H), 1.36 (s, 6H). ^13^C-NMR (125 MHz, CDCl_3_) δ 156.7, 148.3, 142.5, 141.9, 138.3, 130.6, 129.7, 124.4, 121.8, 14.7, 14.6. ^11^B-NMR (160 MHz, CDCl_3_) δ 0.76 (m, BF_2_).

### 3.2. Synthesis of Boronic Acid BODIPY Derivatives 1,2

4-nitrophenyl BODIPY (100 mg, 0.27 mmol) and 4-formylphenylboronic (160 mg, 1 mmol) were dispersed in anhydrous toluene (20 mL) in a vessel saturated with argon, then piperidine (1 mL) and glacial acetic acid (1 mL) were added to the mixture. Microwave irradiation was applied in the appropriate apparatus supplied with Liebig’s condenser and the maximum power was maintained to favor a vigorous boil for 6 h. The solution turned dark blue after a few dozen minutes and the TLC analysis shows two purple and blue spots with Rf 0.6 and 0.2, respectively, using silica gel aluminium plate and 10% methanol/dichloromethane as an eluent. The solvent was removed by roto-evaporation and the solid residue was purified by a chromatographic column loaded with silica gel and eluted with a gradient from 5% methanol/dichloromethane to 30% methanol/dichloromethane. Three fractions were separated in the following order: unreacted BODIPY (18 mg, yield 19%), monostyryl BODIPY **1** (42 mg, yield 30%) as purple solid and distyryl BODIPY **2** (65 mg, yield 38%) as blue solid.

#### 3.2.1. Styryl Boronic Acid BODIPY 1

^1^H-NMR (500 MHz, CDCl_3_) δ 8.39 (d, J = 8.8 Hz, 2H), 7.57 (d, J = 8.0 Hz, 2H), 7.57 (d, J = 7.4 Hz, 1H), 7.51 (d, J = 8.8 Hz, 2H), 7.22 (d, J = 7.4 Hz, 1H), 6.85 (d, J = 7.4 Hz, 2H), 6.62 (s, CH), 6.03 (s, CH), 2.60 (s, 3H), 1.42 (s, 3H), 1.38 (s, 3H).^13^C-NMR (125 MHz, CDCl_3_) δ 164.7, 154.7, 144.7, 143.2, 143.1, 136.2, 132.1, 130.9, 128.8, 126.8, 124.1, 123.3, 120.8, 118.3, 113.5, 83.8, 48.5, 24.9, 14.7, 14.5.^11^B-NMR (160 MHz, CDCl_3_) δ 32.07 (s, broad, B(OH)_2_), 5.07 (t, J = 34 Hz, BF_2_). Molecular weight: 501.10. Anal. calcd. for C_26_H_23_B_2_F_2_N_3_O_4_: C, 62.32; H, 4.63; B, 4.31; F, 7.58; N, 8.39; O, 12.77%. Found: C, 62.29; H, 4.64; B, 4.35; F, 7.57; N, 8.42; O, 12.75%.

#### 3.2.2. Distyryl Boronic Acid BODIPY 2

^1^H-NMR (500 MHz, CD_3_OD) δ 8.42 (d, J = 8.4 Hz, 2H), 7.97 (d, J = 8.0 Hz, 4H), 7.87 (d, J = 7.9 Hz, 2H), 7.85 (d, J = 8.0 Hz, 4H), 7.83 (d, J = 8.4 Hz, 2H), 7.60 (d, J = 7.9 Hz, 2H), 7.05 (s, 2H), 1.41 (s, 6 H)^. 13^C-NMR (125 MHz, CD_3_OD) δ 165.6, 153.0, 142.2, 138.2, 137.6, 135.3, 132.7, 130.9, 129.1, 127.7, 126.7, 124.8, 123.9, 122.8, 114.60, 19.10. ^11^B-NMR (160 MHz, CD_3_OD) δ 31.8 (s, broad, B(OH)_2_), 5.06 (t, J = 33 Hz, BF_2_). Molecular weight: 633,02. Anal. calcd. for C_33_H_28_B_3_F_2_N_3_O_6_: C, 62.61; H, 4.46; B, 5.12; F, 6.00; N, 6.64; O, 15.16%. Found: C, 62.57; H, 4.48; B, 5.09; F, 6.04; N, 6.61; O, 15.19%.

### 3.3. Preparation of TiO_2_ Anodes

Platinum counter-electrodes and DSSC assembly FTO-glass substrates were cleaned in detergent and washed with water/ethanol solution. Then, the cleaned FTO-glass was pre-treated by immersion in 100 mL TiCl_4_/water solution (40 mM) at 70 °C for 30 min, washed again with water and ethanol, and finally dried in an oven at 80 °C. TiO_2_ layers were deposited on the FTO-conducting glass plates by a screen-printing (43.80 mesh/cm, polyester fibers, snap off 0.8 mm) procedure (coating, storing in ethanol and drying at 125 °C), which was repeated several times depending on the required TiO_2_ thickness. Then, the prepared TiO_2_-coated plates were slowly preheated at a temperature of 325 °C for 5 min, which was gradually increased to 375 °C, and sintered at 500 °C for 30 min, as described in [3]. After sintering, the resulting mesoscopic oxide film was transparent, with a thickness ranging around 12 μm, depending on the number of TiO_2_-deposited layers (measured by a BRUKER, Detakk profilometer) and a surface area of each spot of 0.196 cm^2^. After size measurements, the TiO_2_ film was treated with 40 mM TiCl_4_ solution, as described above, rinsed with water, and ethanol and sintered at 500 °C. The cooled screen-printed plates, cut in rectangular forms (2 cm × 2 cm), were soaked overnight in the dye solution at room temperature in the dark. Finally, after dye excess was removed by rinsing with ethanol, the photo-anodes were dried in an oven (at 80 °C for short time) and stored in a desiccator at room temperature for later use. The photoanodes for UV-Vis absorption measurements were carried out using a doctor-blade technique to obtain a transparent, thin TiO_2_ film with an estimated thickness (after the sintering process) of ~10 µm and an active area of 3 cm^2^ (3 cm × 1 cm). To prepare the CEs, a hole was drilled (1.0 mm diameter) in each FTO-glass plate (2 cm × 2 cm). The perforated substrates were washed and cleaned with water and ethanol to remove any residual of glass powder and/or organic contaminants. The Pt catalyst was deposited on the conductive face of the FTO glass by dropping H_2_PtCl_6_ solution (5 mM in isopropanol) and heating at 500 °C for 30 min. The cells, prepared by placing the WE and the Pt-CE in a sandwich-type arrangement with the electrolyte in between, were sealed using a thermo-press with a hot melt gasket of 25 nm thickness, made of the Surlyn ionomer (Solaronix, Aubonne, Switzerland). The aperture of the surlyn frame was larger than the TiO_2_ area. Two kinds of solution were used as electrolytes: acetonitrile (0.1 M LiI 0.05 M I_2_, TBP 0.6 M and thiocyanate 0.1 M) and water (composed of quinone and hydroquinone). We realized three devices for each BODIPY dye in an acetonitrile-based electrolyte and two devices for each BODIPY dye in a water-based electrolyte. In this study, we present the best result for each kind of device, while we report other PV parameters in the Supporting Information.

## 4. Conclusions

The present study is a pioneering work on the synthesis of two new BODIPY dyes and their application in dye-sensitized solar cells. This study also aimed to analyze the photo-physical and photochemical properties of two new BODIPYs **1** and **2,** used as sensitizers for DSSC. The molecules exhibit the boronic function (B(OH)_2_) as an anchoring group to TiO_2_. A nitro group in the phenyl meso-position was inserted to allow for future chemical derivatizations through a reduction to amine, which will favour any addition. In the design of effective sensitizers, the choice of anchor group is extremely important because it can influence both the stability of the connection and the electronic coupling between the dye and the semiconductor. In particular, the presence of multiple boronic functions increases the stability of the dye on the semiconductor and increases the photo-current efficiency. The double anchoring on TiO_2_ by boronic groups could favour a greater rigidity in the dye and, thanks to its shorter distance, it could facilitate the electronic transfer from the BODIPY to TiO_2_. All the investigated species show interesting luminescence in solution and solid matrix. 

To our knowledge, this is the first study that presents a water-based BODIPY DSSC system and successfully demonstrates that this system is also able to convert light energy in electrical energy, opening a new scenario and moving toward the use of environmentally friendly biomimetic solar cells.

## Figures and Tables

**Table 1:** Photophysical properties of BODIPYs **1** and **2**.

	Absorption ^a^		Luminescence				
Sample			289 K ^a^			77 K ^b^	
	λ_max_ (nm)	ε (M^−1^cm^−1^)	λ_max_ (nm)	τ (ns)	Φ	λ_max_ (nm)	τ (ns)
BODIPY **1**	328 526 564	29,000 20,000 68,000	580	0.8	0.008	575	4.3
BODIPY **2**	351 585 633	112,000 43,000 116,000	650	1.8	0.10	645	4.0

^a^ acetonitrile at 298 K, ^b^ butyronitrile in a solid matrix at 77 K.

**Table 2:** Redox data of BODIPY **1** and **2**.

Sample	E_rid/_V vs. SCE	E_ox_/V vs. SCE
BODIPY **1**	−0.903	1.04
BODIPY **2**	−0.857	0.959

All data are recorded in acetonitrile solution.

**Table 3:** Photovoltaic performances of the best-realized device for BODIPY **1** and BPDIPY **2**.

	V_OC_ (V)	J_SC_ (mA/cm^2^)	FF (%)	PCE (%)	IPCE (%)	I-Jsc
BODIPY **1**	0.267	0.73	55	0.11	4	0.78
BODIPY **2**	0.377	3.10	54.9	0.63	24	2.7

Cell performance is measured under solar simulator lighting (95 mW/cm^2^), TiO_2_ thickness: 12 μm; active area: 0.196 cm^2^. In acetonitrile; I^−^/I^3−^ used as mediator.

**Table 4:** Photovoltaic performance of the best-realized BODIPY **1** and **2** devices with the water-based electrolyte.

	V_OC_ (V)	J_SC_ (mA/cm^2^)	FF (%)	PCE (%)
BODIPY **1**	0.057	0.0237	28.4	0.0004
BODIPY **2**	0.128	0.0195	38.8	0.0010

The performance of the cells is measured under lighting with a solar simulator (95 mW/cm^2^), thickness of TiO_2_: 12 μm; active area: 0.196 cm^2^. In water using H_2_Q/Q as a mediator.

## Data Availability

Not applicable.

## Supplementary Materials

The following are available online at https://www.mdpi.com/article/10.3390/biomimetics7030110/s1, Figure S1: DSSC device with BODIPY 1 and DSSC device with BODIPY 2, Figure S2: 1H-NMR (500 MHz, CDCl3) of 8-(p-nitrophenyl)-3,5,7,9-tetramethyl-BODIPY, Figure S3: 13C-NMR (125 MHz, CDCl3) of 8-(p-nitrophenyl)-3,5,7,9-tetramethyl-BODIPY, Figure S4: 11B-NMR (128 MHz, CDCl3) of 8-(p-nitrophenyl)-3,5,7,9-tetramethyl-BODIPY, Figure S5: 1H-NMR (500 MHz, CDCl3) of sStyryl bBoronic aAcid BODIPY 1., Figure S6: 11B-NMR (128 MHz, CDCl3) of sStyryl bBoronic aAcid BODIPY 1., Figure S7: Mass of sStyryl bBoronic aAcid BODIPY 1, Figure S8: 1H-NMR (500 MHz, DMSO-d6) of distyryl bBoronic aAcid BODIPY 2., Figure S9: 13C-NMR (125 MHz, DSMO-d6) of distyryl bBoronic aAcid BODIPY 2, Figure S10: Mass of distyryl boronic acid BODIPY 2.; Figure S11: Differential pulse voltammograms of BODIPY 1 in acetonitrile (scan rate 20mV/s) (Fc= Ferrocene); Figure S12: Differential pulse voltammograms of 1BODIPY 2 in acetonitrile (scan rate 20mV/s) (Fc= Ferrocene).; Figure S13: I-V curve of BODIPY 1, Figure S14: I-V curve of BODIPY 2, Figure S15: Incident photon-to-current conversion efficiency (IPCE) and Integrated current density for the best realized device of (A) BODIPY 1 and (B) BODIPY 2. Table S1: Photovoltaic performances of realized devices for BODIPY 1 and BPDIPY 2. In acetonitrile and I-/I3- as mediator. Table S2: Photovoltaic performances of realized devices for BODIPY 1 and BPDIPY 2. In water and Q/H2Q as mediator.

## Sample Availability

Samples of the BODIPYs **1** and **2** are available from the authors.
