# Supplementary material for: Synthesis and Photophysics Characterization of Boronic Styril and Distyryl BODIPYs for Water-Based Dye-Sensitized Solar Cells
Source: Biomimetics (Basel). 2022 Aug 11;7(3):110. doi: 10.3390/biomimetics7030110 (PMC9397057; doi:10.3390/biomimetics7030110)
Supplement: Supplementary file 1 [file biomimetics-07-00110-s001.zip › biomimetics-1811421-supplementary.pdf]

Table of Contents

1. DSSC with dye 1 and dye 2……………………………………………………..S1

2. NMR spectra (^{1}H, ^{13}C, ^{11}B) and mass spectra……………………….S2-S10

3. Additional data……………….……………………………………………...... S11-S13

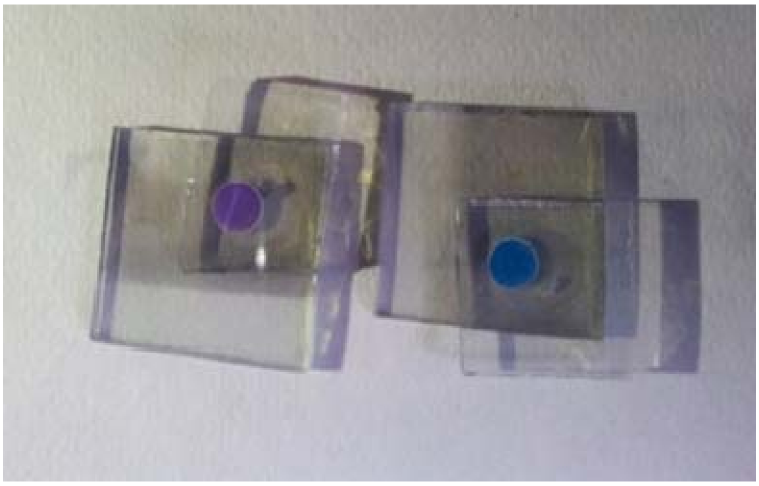

Figure S1. *DSSC device with BODIPY **1** and DSSC device with BODIPY **2**.*

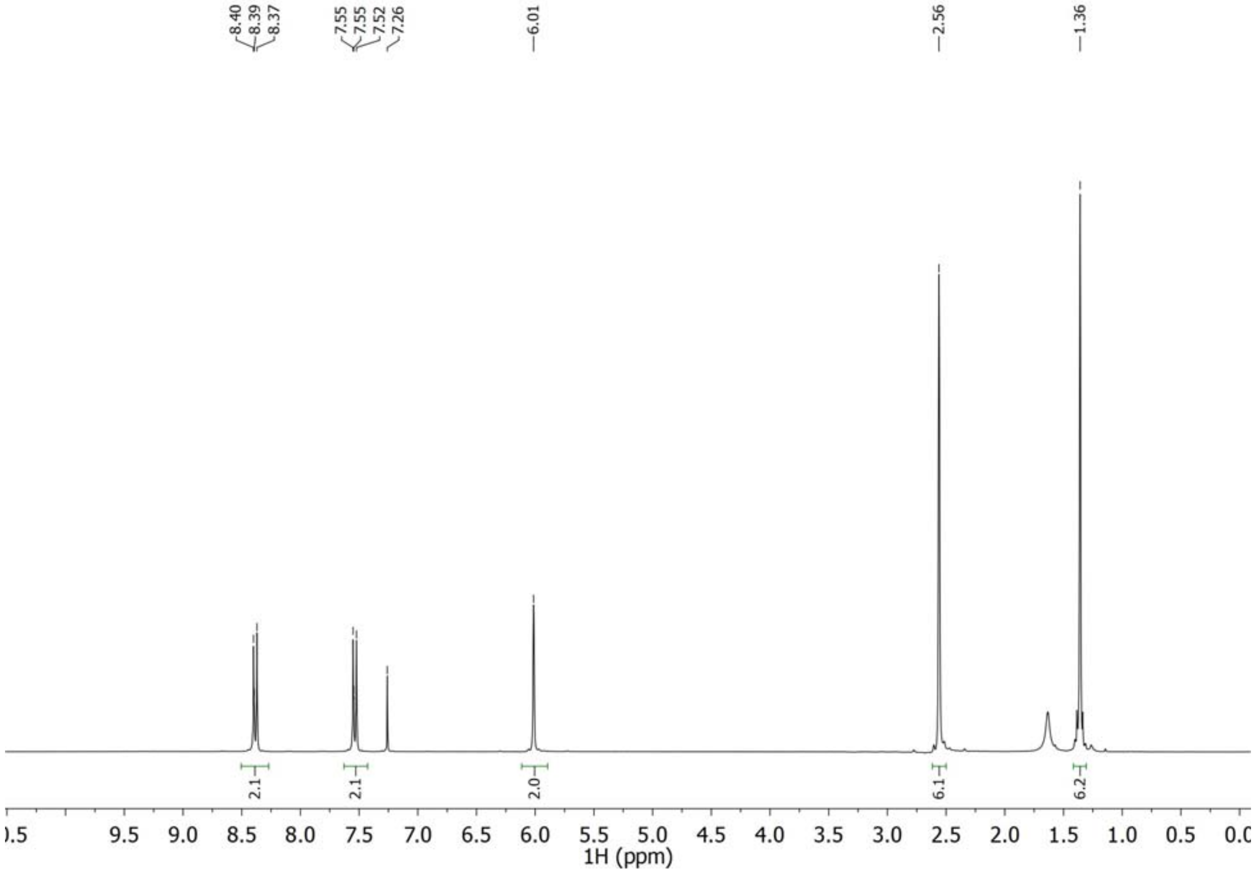

Figure S2. *^{1}H-NMR (500 MHz, $CDCl_3$) of 8-(p-nitrophenyl)-3,5,7,9-tetramethyl-BODIPY.*

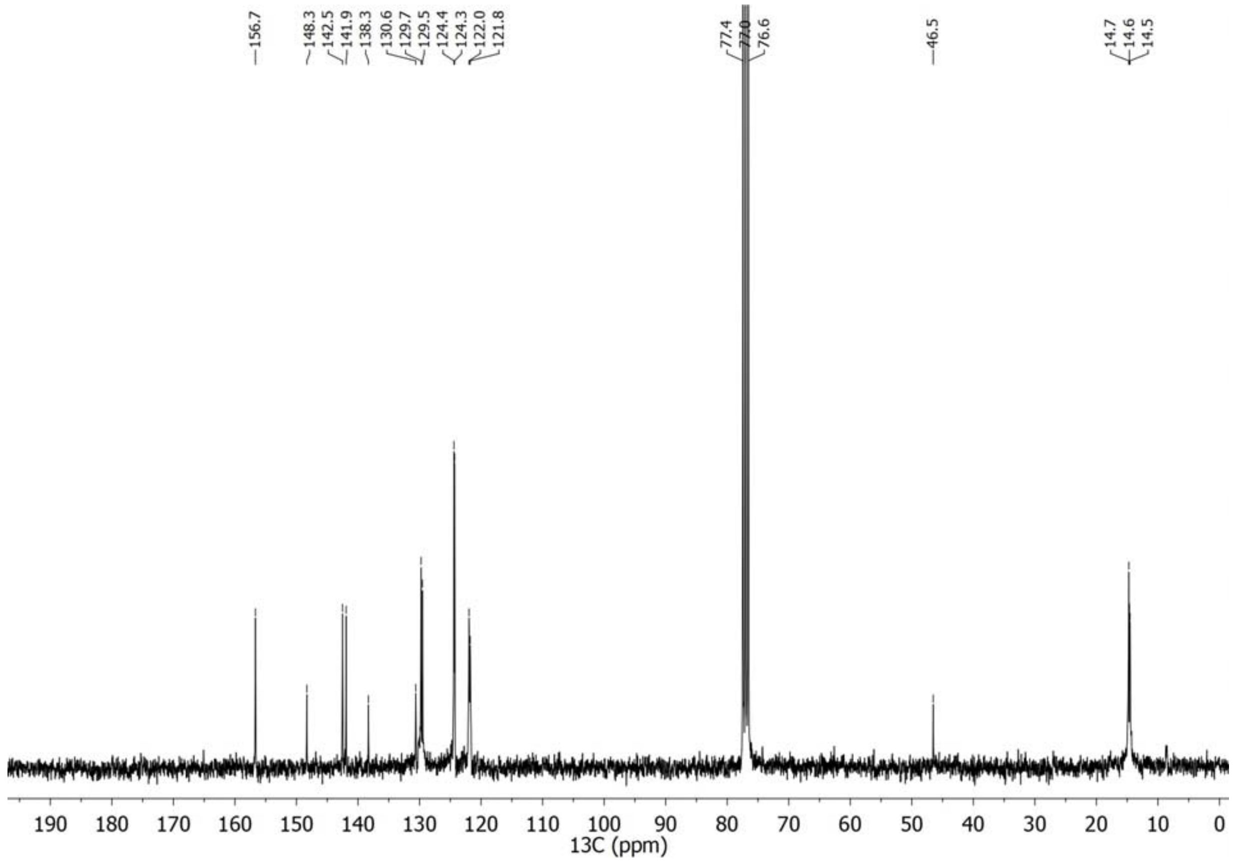

Figure S3. *^{13}C-NMR (125 MHz, $CDCl_3$) of 8-(p-nitrophenyl)-3,5,7,9-tetramethyl-BODIPY.*

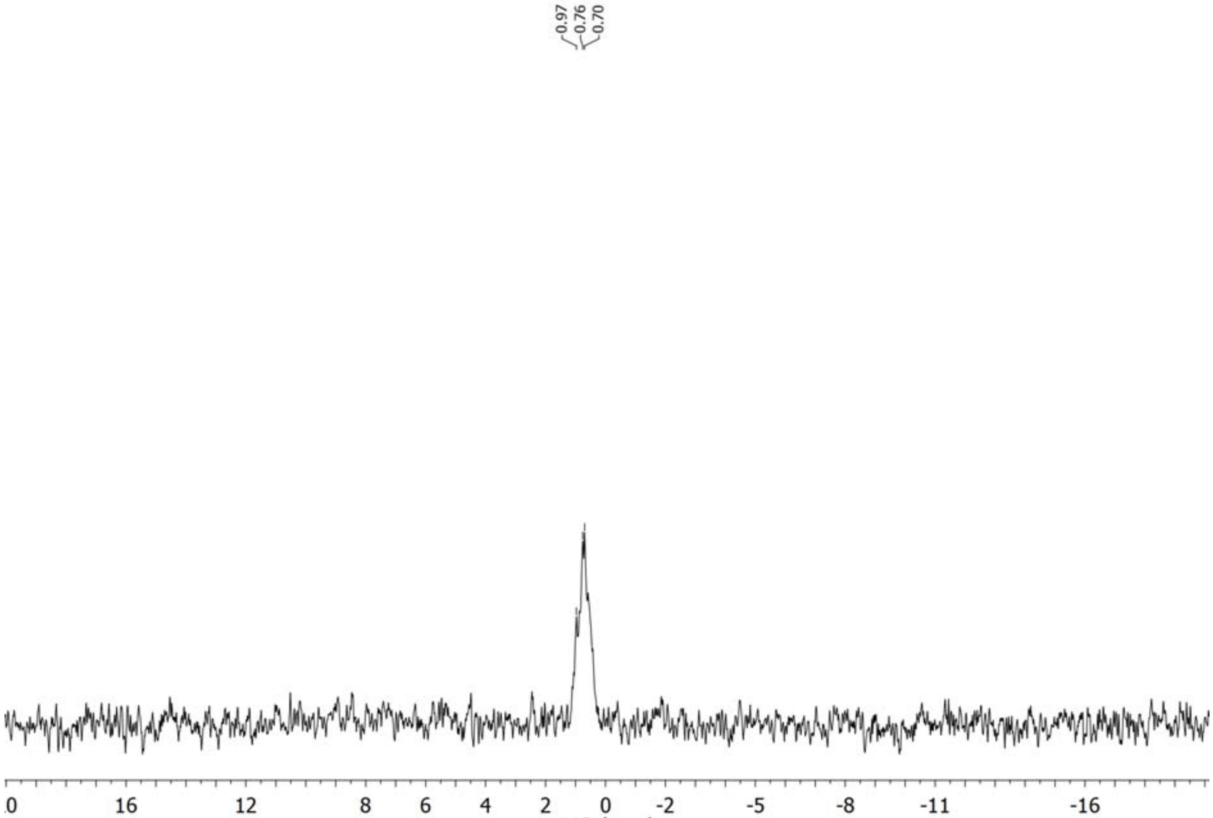

Figure S4. *^{11}B-NMR (128 MHz, $CDCl_3$) of 8-(p-nitrophenyl)-3,5,7,9-tetramethyl-BODIPY.*

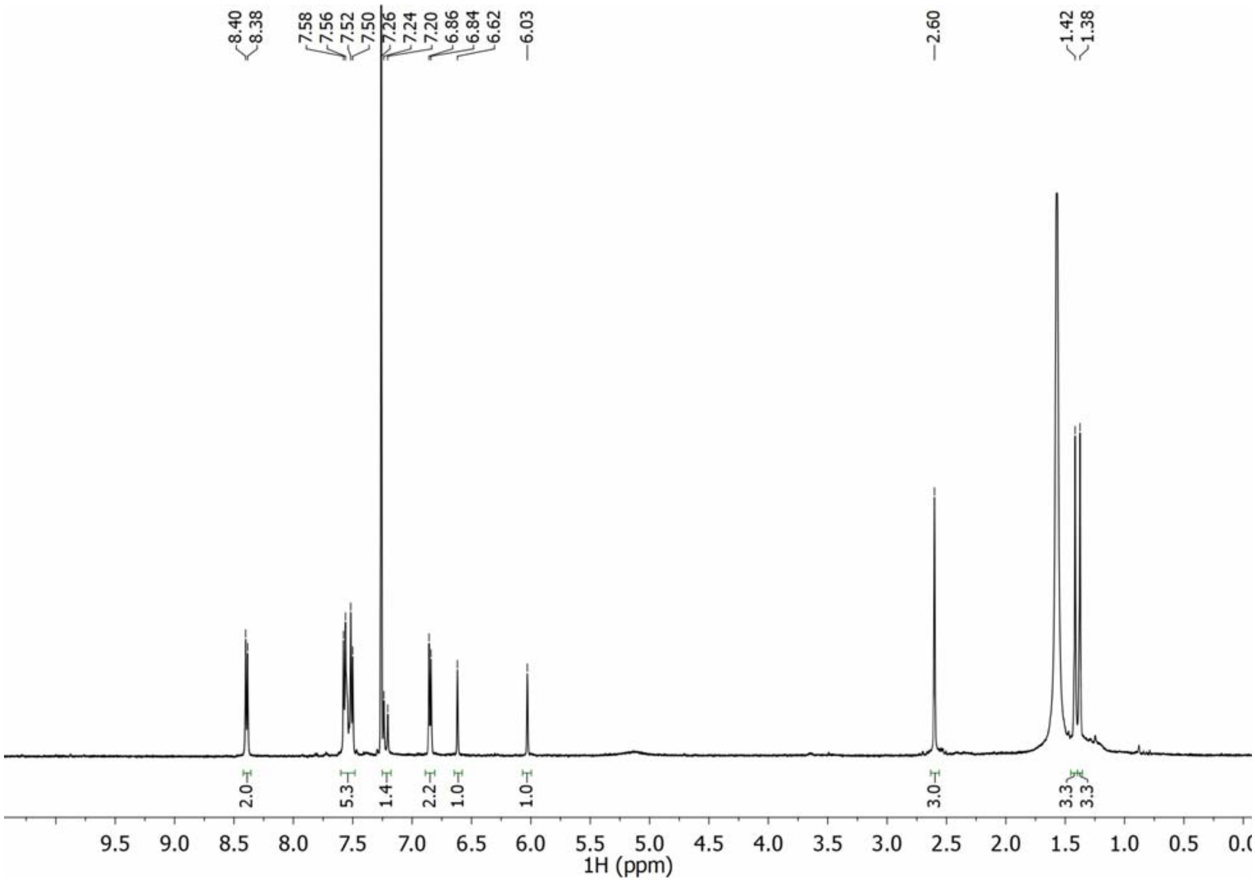

Figure S5. *^{1}H-NMR (500 MHz, $CDCl_3$) of styryl boronic acid BODIPY **1.***

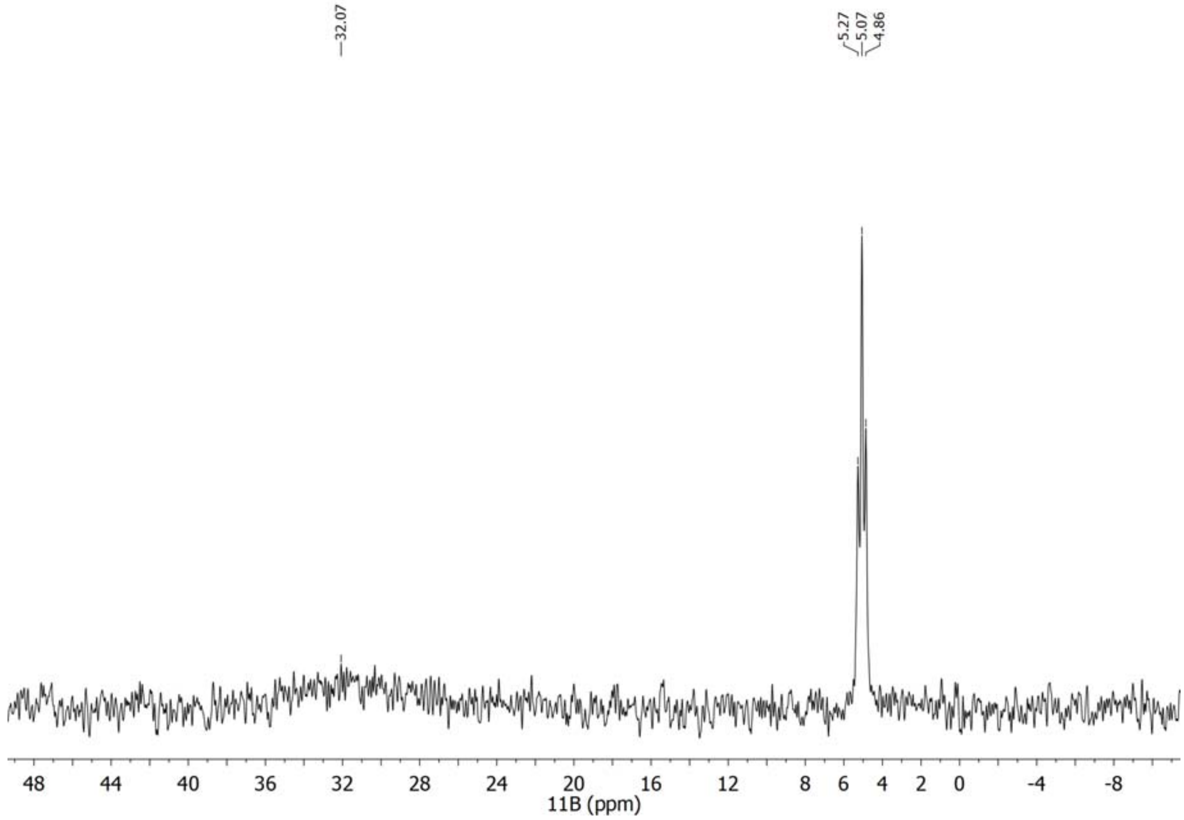

Figure S6. *^{11}B-NMR (128 MHz, $CDCl_3$) of styryl boronic acid BODIPY **1.***

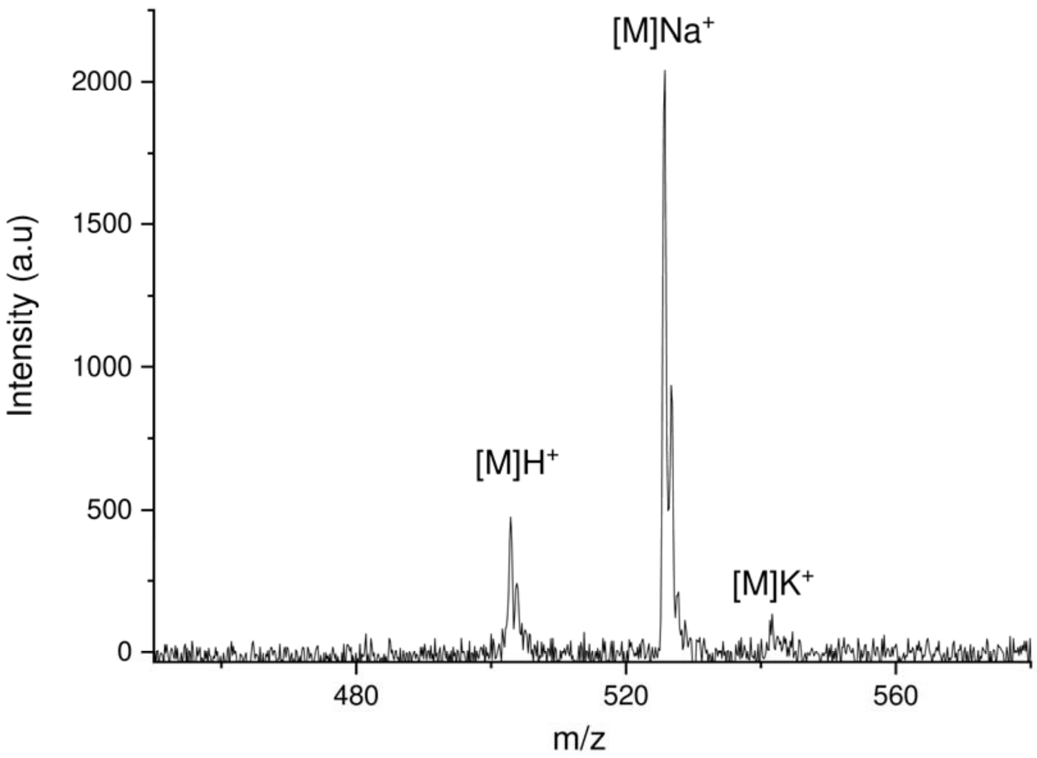

Figure S7. *Mass of styryl boronic acid BODIPY **1**.*

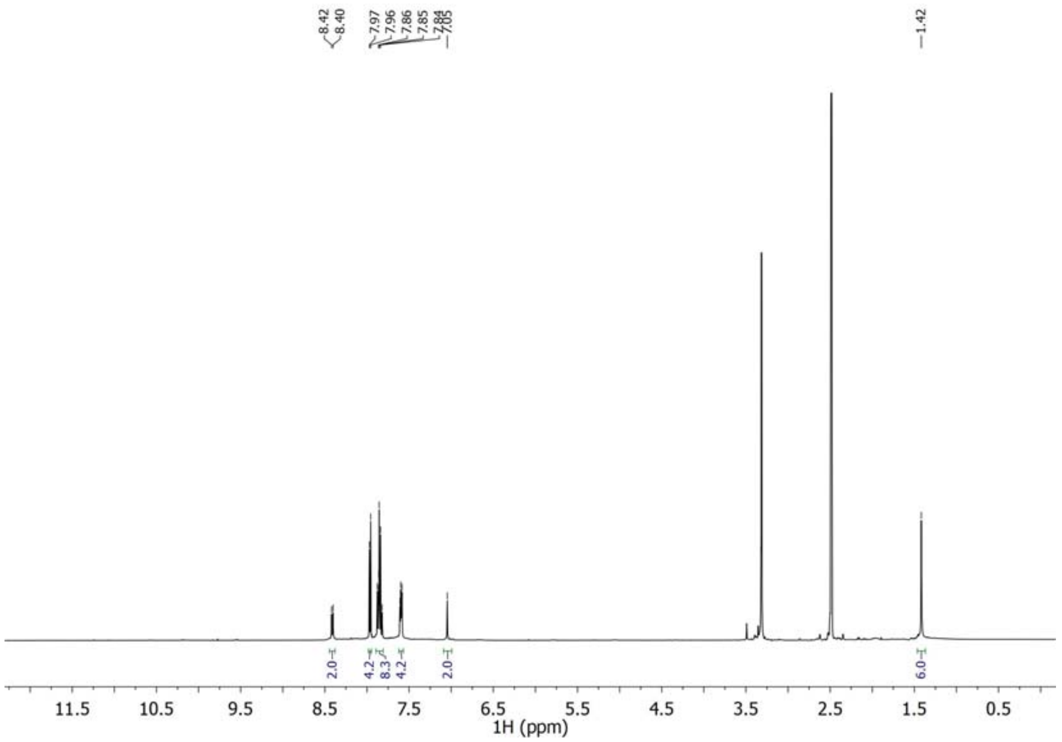

Figure S8. *^{1}H-NMR (500 MHz, DMSO-d6) of distyryl boronic acid BODIPY **2**.*

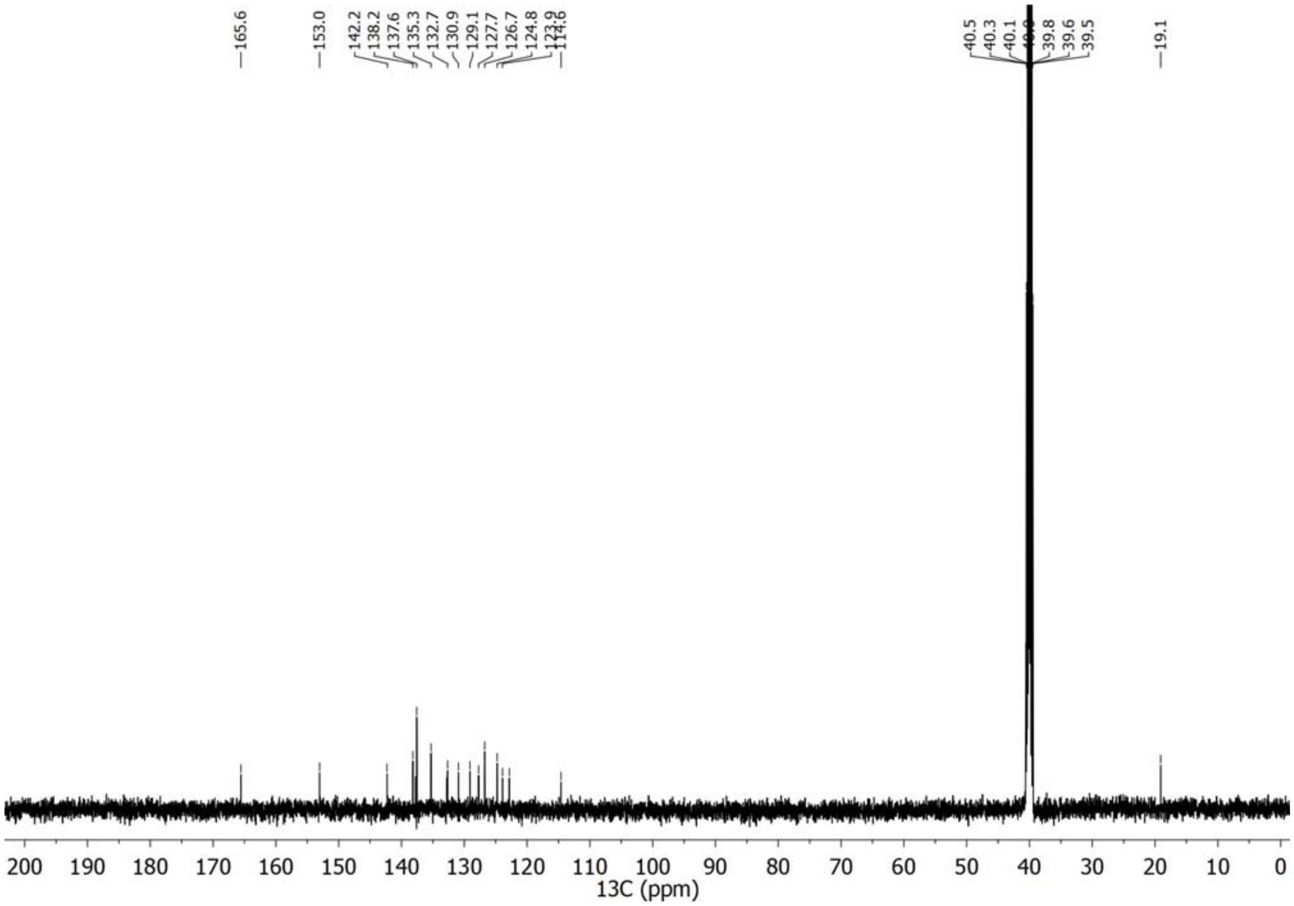

Figure S9. *^{13}C-NMR (125 MHz, DSMO-d$_6$) of distyryl boronic acid BODIPY **2.***

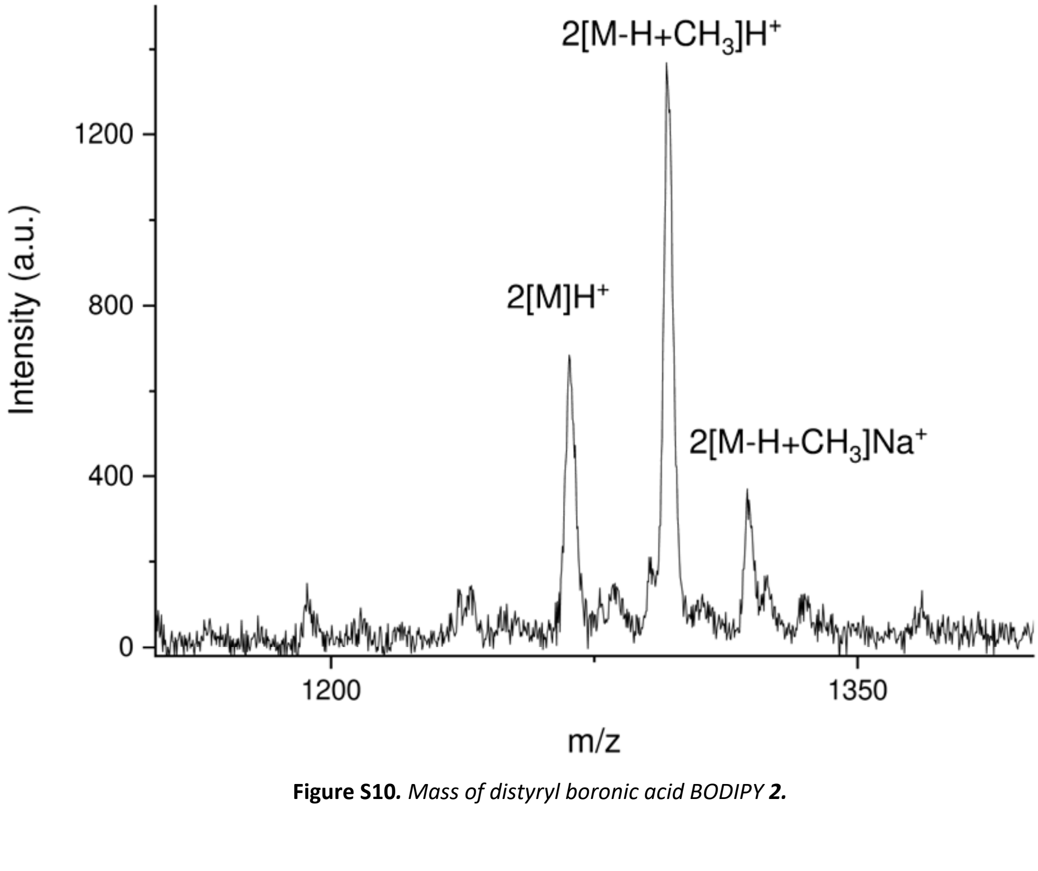

Figure S10. *Mass of distyryl boronic acid BODIPY **2.***

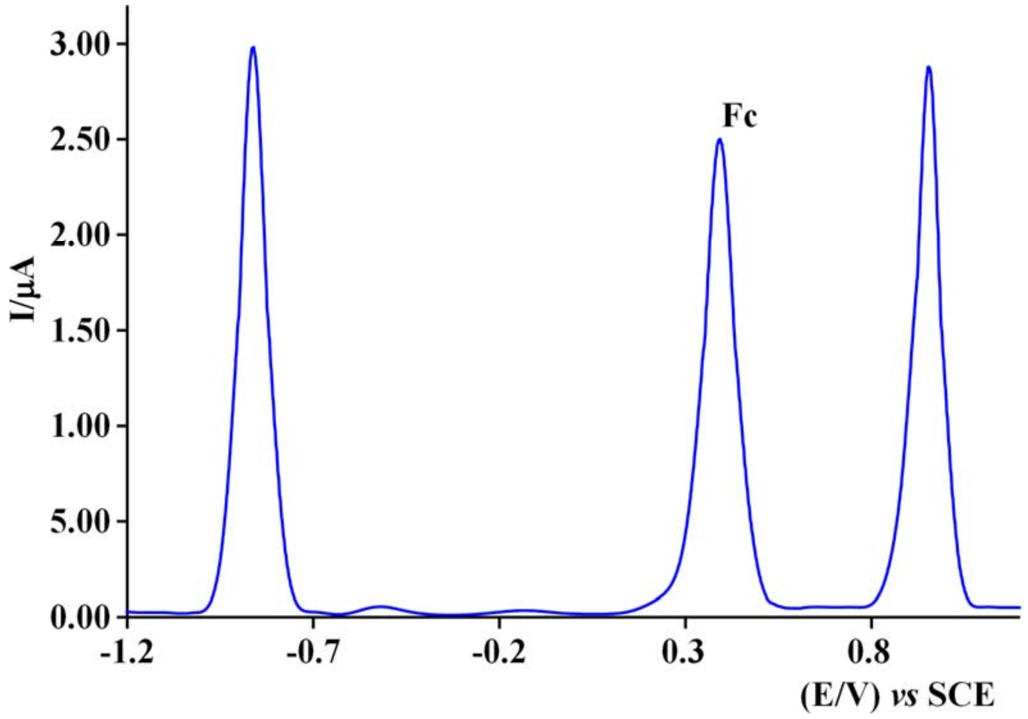

Figure S11. Differential pulse voltammograms of BODIPY 1 in acetonitrile (scan rate 20mV/s) (Fc= Ferrocene).

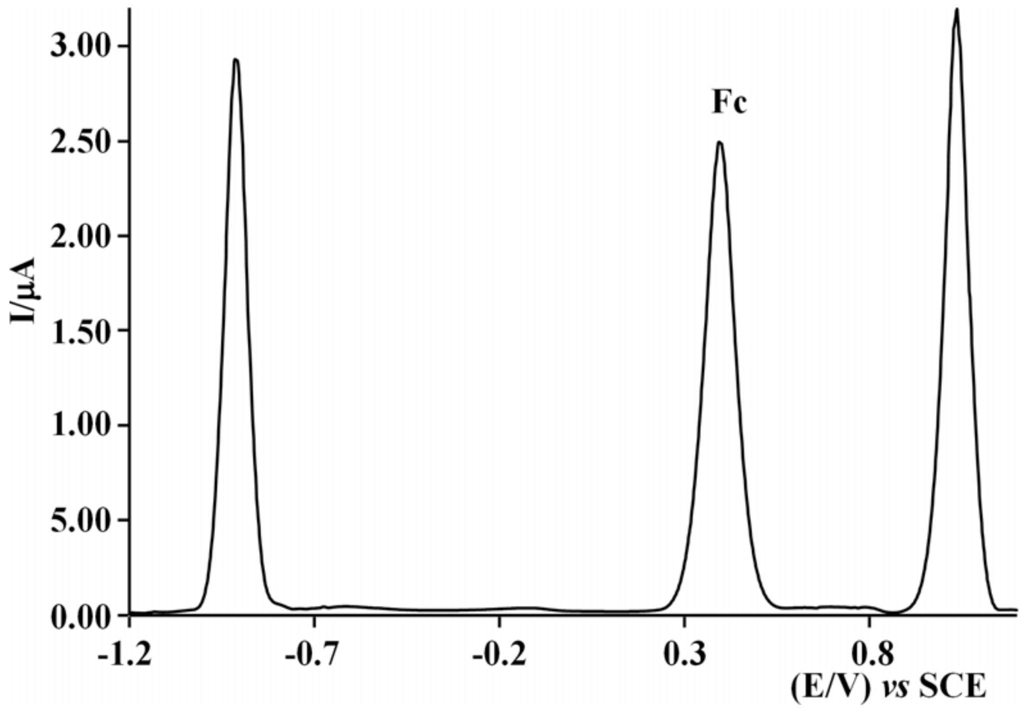

Figure S12. *Differential pulse voltammograms of 1BODIPY 2 in acetonitrile (scan rate 20mV/s) (Fc= Ferrocene).*

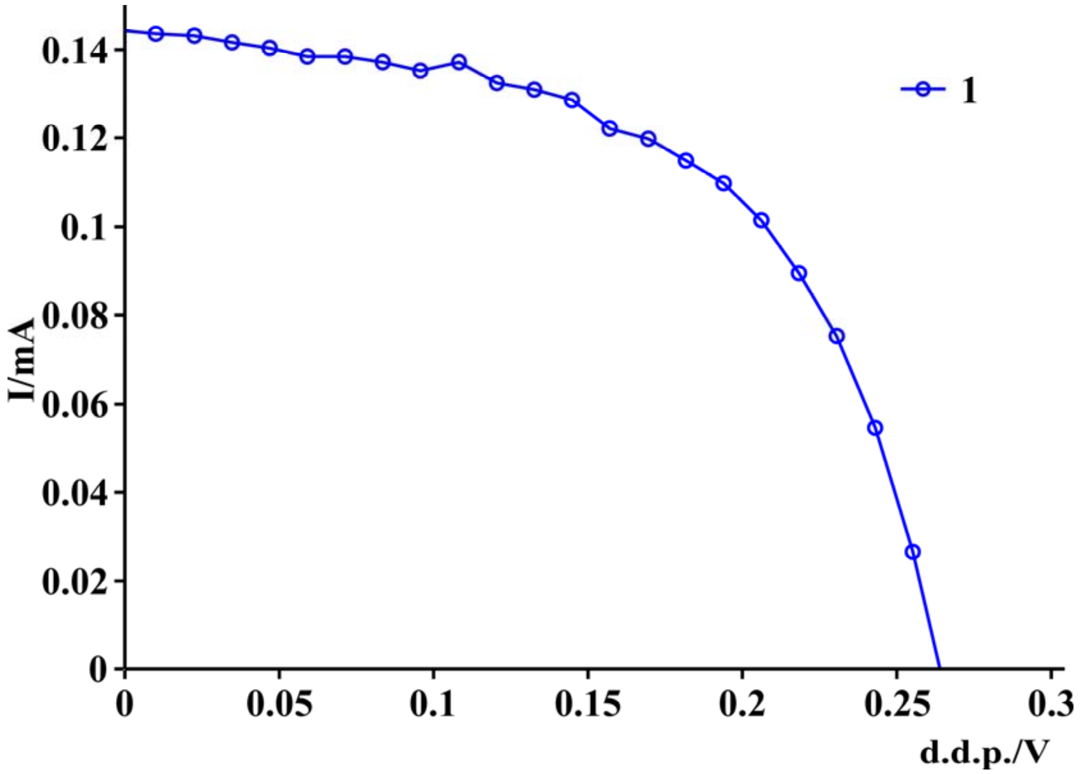

Figure S13. *I-V curve of BODIPY **1**.*

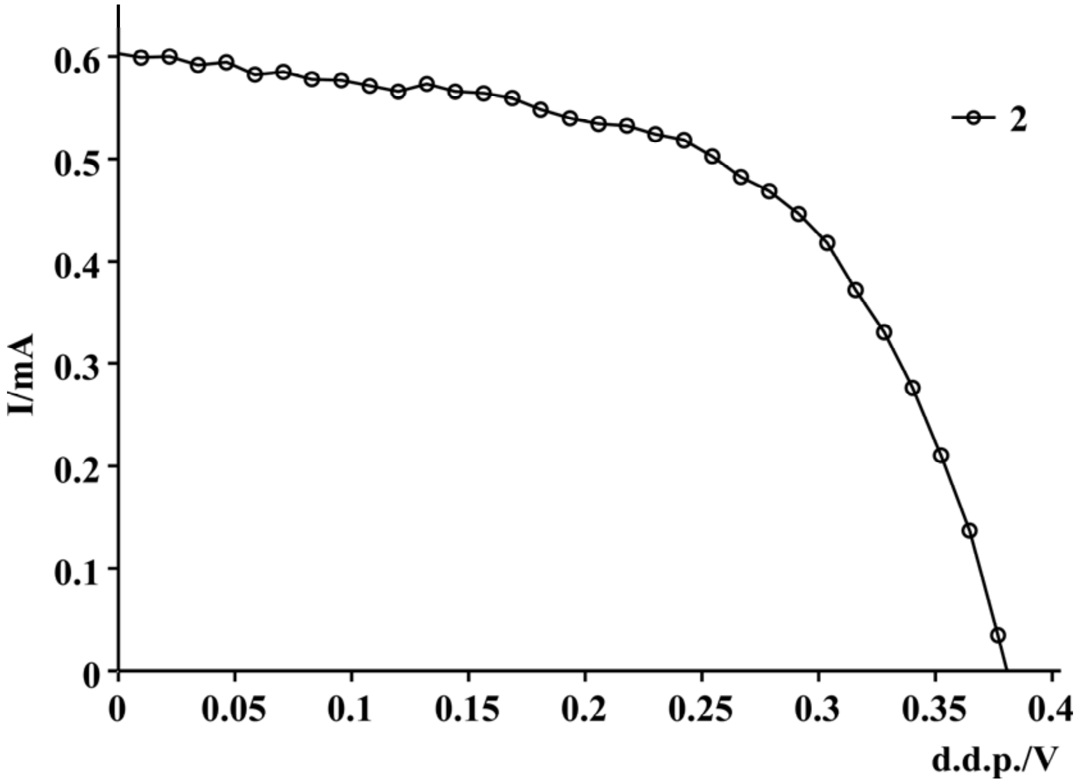

Figure S14. *I-V curve of BODIPY **2**.*

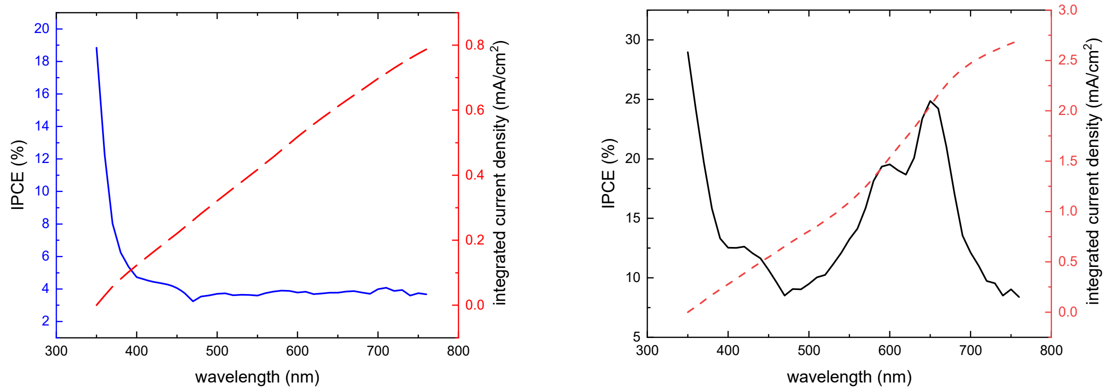

(A) (B)

Figure S15. Incident photon-to-current conversion efficiency (IPCE) and Integrated current density for the best realized device of A) BODIPY 1 and B) BODIPY 2

Table S1. Photovoltaic performances of realized devices for BODIPY 1 and BPDIPY 2. In acetonitrile and I^-/I^{3-} as mediator.

	PCE (%)	Jsc (mA/cm^2)	Voc (V)	FF (%)
BODIPY 1	0.08	0.78	0.21	51
BODIPY 1	0.08	0.46	0.28	62
BODIPY 2	0.4	2.53	0.29	53
BODIPY 2	0.3	1.28	0.30	57

Table S2. Photovoltaic performances of realized devices for BODIPY 1 and BPDIPY 2. In water and Q/H_2Q as mediator.

	PCE (%)	Jsc (mA/cm^2)	Voc (V)	FF (%)
BODIPY 1	0.0004	0.0237	0.057	28.4
BODIPY 1	0.0001	0.018	0.014	34
BODIPY 2	0.00101	0.0194	0.128	38.5
BODIPY 2	0.00102	0.0195	0.128	38.8
